# Adaptive Resonance Demodulation for Bearing Fault Diagnosis via Spectral Trend Reconstruction and Weighted Logarithmic Energy Ratio

**DOI:** 10.3390/s26072066

**Published:** 2026-03-26

**Authors:** Qihui Feng, Yongqi Chen, Qinge Dai, Jun Wang, Jiqiang Hu, Linqiang Wu, Rui Qin

**Affiliations:** 1 College of Science and Technology, Ningbo University, Ningbo 315211, China; 2411170019@nbu.edu.cn (Q.F.); ziliu7781@163.com (Q.D.); takeinvain@163.com (J.W.); 2 Ningbo Donghuang Bearing Co., Ltd., Ningbo 315318, China; xsbearing@126.com (J.H.); 13951577868@139.com (L.W.); qr87@163.com (R.Q.)

**Keywords:** rolling bearing, fault diagnosis, spectral trend reconstruction, weighted logarithmic energy ratio (WLER), adaptive resonance demodulation

## Abstract

Incipient fault signatures in rolling bearings are often compromised by intense background noise and stochastic impulses. Conventional resonance demodulation frequently relies on rigid frequency partitioning, which tends to disrupt the physical continuity of resonance bands and results in the incomplete capture of essential diagnostic information. Furthermore, the robustness of prevailing optimal demodulation frequency band (ODFB) selection indicators remains limited under heavy noise interference. This study develops the WLERgram framework, which utilizes regularized Fourier series to capture the global morphology of the vibration spectrum. By anchoring filter boundaries at natural energy troughs, the method mitigates spectral truncation based on inherent signal characteristics. The framework integrates an Adaptive Morphological Consensus (AMC) strategy, employing multi-scale operators to extract rotation-correlated components and enhance resistance to incoherent interference. By incorporating a Weighted Logarithmic Energy Ratio (WLER) metric, the method utilizes a nonlinear operator to implement differential mapping between coherent fault harmonics and stochastic noise, enabling autonomous optimization of the demodulation band. Validations using synthetic simulations and experimental benchmarks (CWRU and UORED) suggest that WLERgram offers reliable feature extraction performance and diagnostic robustness under harsh noise environments.

## 1. Introduction

Rolling bearings play a central role in bearing loads and transmitting power within rotating machinery [1,2]. Despite their precision design, prolonged cyclic operation under harsh conditions inevitably leads to wear or fatigue failure, posing significant safety hazards [3,4]. To prevent unexpected shutdowns and economic losses resulting from such failures [5,6], research into precise early-stage fault diagnosis technologies has become an urgent industrial need for ensuring production safety and implementing predictive maintenance.

To develop effective diagnostic methodologies for rolling element bearings, it is essential to first understand their fault mechanisms from a physical and dynamic perspective. Specifically, Wu et al. [7] systematically reviewed multi-sensor detection technologies, pointing out that local defects on different bearing components (e.g., inner ring, outer ring, and rolling elements) inevitably generate corresponding theoretical defect frequencies, which can be effectively captured by analyzing one-dimensional signals or two-dimensional images from various external and internal sensors. Furthermore, exploring the root physical cause of these signatures, Chen et al. [8] established a stochastic nonlinear dynamic model for defective bearings and explicitly revealed that these characteristic frequencies essentially originate from the transient interactions between the rolling elements and the defective regions. Their dynamic analysis demonstrated that such interactions induce abrupt changes in localized contact stress and excite strong, periodic nonlinear transient impact pulses, with the applied load acting as the dominant factor amplifying these vibrations. This underlying dynamic process dictates that any robust condition monitoring algorithm must be highly sensitive to capturing these weak, dynamically induced transient impulses from heavy background noise.

Rolling bearing fault diagnosis has therefore long been a central focus in rotating machinery health monitoring; however, the impulsive components from early-stage faults are typically extremely faint and easily masked by intense background noise and harmonic interference from other mechanical components, making reliable feature extraction particularly challenging [9]. Extracting these weak, dynamically induced impulses from heavy background noise thus forms the primary motivation for the advanced signal processing algorithms developed in this study. Existing solutions are broadly categorized into two types: data-driven approaches based on artificial intelligence and mechanism-based methods grounded in signal processing [10]. While machine learning (ML) and deep learning (DL) techniques excel in feature recognition, their heavy reliance on high-quality data and limited generalization capabilities under varying operating conditions constrain their widespread application. In contrast, signal processing-based approaches focus on analyzing the modulation phenomena induced by faults. Since bearing faults typically excite structural resonances, identifying resonance bands rich in fault information—specifically selecting the optimal demodulation frequency band (ODFB)—and subsequently performing envelope demodulation analysis has proven to be an exceptionally effective strategy [11].

As pioneering work in this field, Antoni [12,13,14] proposed the Fast Kurtogram. This method utilizes a 1/3-binary tree filter bank to perform multi-level signal decomposition and locates fault frequency bands based on the principle of maximum kurtosis; it has been widely adopted due to its high efficiency. Although Fast Kurtogram demonstrates favorable computational efficiency and diagnostic performance across various scenarios, the kurtosis metric is inherently sensitive to non-Gaussian noise and random impulsive interference, which often leads to the erroneous selection of the ODFB [15]. Furthermore, the rigid spectral segmentation patterns can result in the fragmentation of resonance bands during the filtering process, thereby reducing the accuracy of fault feature extraction [16]. Consequently, optimization efforts surrounding the Kurtogram have primarily focused on two core issues: the innovation of evaluation metrics and the optimization of frequency band segmentation mechanisms. The former aims to eliminate the influence of random impulses by improving band selection criteria, while the latter attempts to resolve the problem of feature omission caused by fixed boundaries through refined spectral partitioning strategies.

To overcome the inherent limitations of the kurtosis metric, subsequent research has focused on developing more robust statistical evaluation tools. Initially, researchers proposed a series of alternative metrics based on sparsity, such as the spectral smoothness index [17], Hoyer index [18], Gini coefficient [19], and the L_2_/L_1_ norm [20]. However, these metrics share shortcomings similar to kurtosis: their values tend to decrease as the repetition rate of fault impacts increases, thereby compromising diagnostic accuracy. Consequently, to more effectively distinguish periodic fault impacts from random disturbances, academic attention has shifted toward leveraging the cyclostationarity and correlation characteristics of signals. Representative works include the Protrugram proposed by Barszcz et al. [21], which utilizes the kurtosis of the envelope spectrum (ES) instead of time-domain kurtosis, though it may fail under low signal-to-noise ratio (SNR) conditions. Antoni [22] introduced the Infogram, which employs the negative entropy of the square envelope or square envelope spectrum (SES) as the basis for band selection, yet it may perform poorly when noise exhibits non-Gaussian characteristics. Autocorrelation-based methods have also gained favor for their ability to enhance periodic components; for instance, Moshrefzadeh [23] developed the Autogram to identify fault bands via the unbiased autocorrelation kurtosis of narrowband square envelopes, but this method suffers from blurred features under speed fluctuations and high computational overhead. Furthermore, Zhang et al. [24] proposed the ACKgram, which uses iterative adaptive correlation kurtosis to solve prior dependence at the cost of computational efficiency. More recently, Liu et al. [25] proposed the Andgram, which innovatively uses a Variational Autoencoder to fuse multiple indicators into an “anomaly index,” significantly enhancing robustness but introducing high model complexity and parameter-tuning challenges that reduce its interpretability and convenience for engineering applications.

Despite notable progress in frequency band selection indicators, the inherent fixed-band segmentation structure of traditional methods remains a critical bottleneck limiting diagnostic accuracy. The 1/3-binary tree filter bank represented by Fast Kurtogram offers high computational efficiency, yet its rigid geometric divisions cause fragmentation or truncation of resonance bands, dispersing fault energy across multiple sub-bands and significantly degrading the signal-to-noise ratio. To overcome this limitation, recent research has shifted focus toward adaptive spectrum segmentation strategies. Early efforts primarily employed variable-width sliding window mechanisms based on rotational speed to locate fault regions through full-spectrum scanning [26]; however, such approaches heavily rely on prior rotational speed information and suffer from low computational efficiency. Subsequently, data-driven spectral trend reconstruction methods became mainstream: Liu et al. [27] proposed ERFD, which achieves blind segmentation by combining random feature energy spectra with the MHIR metric; Sui et al. [28] introduced the economic Theil index to construct the CTIgram, improving accuracy through multi-level adaptive partitioning. Nevertheless, these methods still require complex iterative optimization or grid search, cannot adaptively determine the number of boundary points, and are susceptible to extreme outliers in the spectrum. These data-driven approaches significantly enhance segmentation flexibility, yet introduce increased computational complexity and stability issues due to iterative optimization and parameter sensitivity. Further morphological trend-based approaches have advanced: Wang et al. [29] developed the Ensefgram using a median-based bisection strategy for blind optimal band selection, while Cai et al. [30] proposed the Encogram to autonomously determine boundaries according to spectral distribution characteristics; unfortunately, both incur extremely high computational resource consumption, limiting their engineering practicality. Ultimately, although existing adaptive FBS methods have addressed the limitations of fixed boundaries, three key challenges remain unresolved: (1) the lack of physically consistent adaptive segmentation boundaries, (2) insufficient robustness in feature extraction under strong noise conditions, and (3) the absence of nonlinear penalty mechanisms in evaluation indicators. Addressing these issues in a unified framework remains an urgent challenge.

To address the limitations of fixed frequency band segmentation in traditional methods and the instability of existing indicators under strong noise, this paper proposes a rolling bearing fault diagnosis method based on spectrum trend adaptive segmentation and weighted logarithmic energy ratio, termed WLERgram. The method first reconstructs the global energy topology of the spectrum using regularized Fourier modeling and utilizes the natural troughs of the trend curve as physical boundaries for frequency segmentation. By integrating these boundaries into a 1/3-binary tree structure, an adaptive filter bank is constructed to achieve hierarchical segmentation, effectively preventing the unintended truncation of fault resonance bands. Building upon this foundation, an Adaptive Morphological Consensus (AMC) strategy is proposed to precisely lock onto target fault features, and a robust statistical metric—the Weighted Logarithmic Energy Ratio (WLER)—is established to identify the ODFB. Finally, fault diagnosis is completed through ES analysis of the optimized band. The main contributions and innovations of this paper are as follows:

(1) The WLERgram diagnostic framework is proposed. This method utilizes spectral trend reconstruction theory to achieve adaptive spectrum segmentation. It effectively overcomes the inherent defects of traditional fixed-band partitioning methods (such as Fast Kurtogram) that often compromise the integrity of broadband resonance features, thereby ensuring the lossless extraction of physical characteristics.
(2) The WLER evaluation metric is constructed. This metric deeply integrates the AMC feature extraction strategy with a nonlinear logarithmic penalty mechanism. Without requiring prior fault knowledge, it can effectively suppress random noise interference and precisely quantify the richness of fault information within each narrowband signal.
(3) Comprehensive validation of performance. Through simulation analysis and experimental verification, it is demonstrated that WLERgram possesses high accuracy and robustness in extracting weak fault features under strong noise interference and speed fluctuation conditions. Its comprehensive performance is shown to be superior to existing mainstream methods.

The organization of this paper is as follows: Section 2 details the core theoretical framework of WLERgram, including the spectral trend adaptive segmentation mechanism based on regularized Fourier series, the feature extraction strategy based on AMC, and the construction principles of the WLER indicator. Section 3 summarizes the specific implementation steps of the method and provides a complete algorithm flowchart. In Section 4, the capability of the method to extract faint fault features under complex conditions is validated by constructing synthetic simulation signals that incorporate strong background noise, random pulses, and discrete interference, followed by a comparative analysis with other mainstream methods. Section 5 further verifies the effectiveness and robustness of the proposed method in practical engineering scenarios using inner-ring fault data from Case Western Reserve University (CWRU) and no-load rolling-element fault data from the University of Ottawa (UORED). Finally, Section 6 summarizes the research conclusions and outlines future research directions.

## 2. Proposed Fault Diagnosis Method: WLERgram

To address the limitations of traditional fixed-band segmentation methods under intense noise and to enhance the robustness of indicators against rotational speed fluctuations and random impulses, this paper proposes a novel method termed WLERgram. The method comprises three core steps: adaptive frequency band segmentation based on spectral trend reconstruction, target feature extraction utilizing an AMC strategy, and optimal band selection guided by the WLER.

### 2.1. Adaptive Spectrum Segmentation Based on Spectral Trends

**Figure 1:**
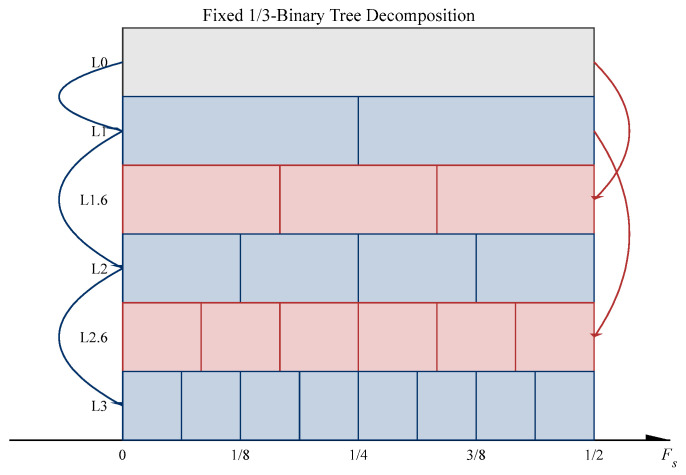
The 1/3-binary tree structure.

The classical 1/3-binary tree filter bank, as employed in the Fast Kurtogram, adopts a “blind” segmentation strategy. As illustrated in Figure 1, it forcibly partitions the frequency axis according to fixed geometric ratios (e.g., at 1/2 or 1/3 intervals). This rigid segmentation possesses significant physical deficiencies:

(1) Feature Truncation: The resonance bands excited by faults occur at random spectral locations. If a resonance band happens to align with a theoretical split point, the fixed boundary will “bisect” the complete resonance peak. This causes the feature energy to disperse into two adjacent sub-bands, significantly degrading the SNR.
(2) Noise Retention: Fixed bandwidths often fail to compactly envelop the resonance zone. Consequently, sub-bands may contain excessive non-resonant frequency components, which dilutes the fault signatures and hinders effective feature extraction.

To overcome the aforementioned deficiencies, it is imperative to establish a mechanism capable of perceiving the “valleys” and “peaks” of the spectral energy distribution. This ensures that the segmentation boundaries automatically bypass resonance peaks (high-energy regions) and instead align with spectral troughs (low-energy regions).

#### 2.1.1. Spectral Trend Extraction Based on Overcomplete Fourier Series

To extract a smooth and physically meaningful energy profile from the Fourier spectrum S(f) containing strong random noise, this paper models it as a linear combination of an orthogonal basis function set [31]. To balance computational efficiency and global fitting accuracy, the frequency axis is mapped to the normalized interval $t_{norm}\in \left[0,1\right]$, and a regression matrix K based on an overcomplete Fourier series is constructed:

(1) $$K=\left[1,\cos\left(\pi \cdot j\cdot t_{norm}\right),\sin\left(\pi \cdot k\cdot t_{norm}\right)\right]\;\left(j,k=1,\ldots ,M\right),$$

where j and k denote the orders of the cosine and sine basis functions, respectively, both taking integer values from 1, 2, …, *M*. *M* represents the highest order controlling the fitting precision. $t_{norm}$ is a normalization variable introduced for numerical stability, mapping the original frequency axis f to the interval [0, 1].

To prevent overfitting caused by random noise in the spectrum, this paper adopts a Tikhonov regularization framework, smoothing trend terms by penalizing the magnitude of the coefficient vector c. The optimal solution is obtained by minimizing the sum of squared residuals and the regularization term [32]:

(2) $$c^{*}={\left(K^{T}K+\lambda I\right)}^{-1}K^{T}S_{fit},$$

where I denotes the identity matrix, and ${\left(\cdot \right)}^{-1}$ represents the matrix inverse operation. Ultimately, a smooth spectral trend line $S_{t}=Kc^{*}$ is obtained through linear reconstruction. In this paper, λ (set to 0.01) is a tuning parameter, and $S_{fit}$ is the downsampled spectrum after linear interpolation to enhance the processing speed. The reconstructed smooth spectral trend line $S_{t}$ can be expressed as:

(3) $$S_{t}=Kc^{*}.$$

#### 2.1.2. Construction of Adaptive 1/3-Binary Tree Filter Banks

The set of local minima points $\Omega_{bound}=\{b_{1},b_{2},\ldots ,b_{P}\}$ on the spectral trend curve $S_{t}$ forms the natural boundary of the spectral energy bulge region. This paper does not directly abolish the hierarchical decomposition logic of the 1/3-binary tree but instead employs a “Nearest Neighbor Correction” strategy to optimize it.

Specifically, the proposed method retains the fixed number of sub-bands at each decomposition level k inherent to the standard 1/3-binary tree. The adaptability of WLERgram lies exclusively in dynamically shifting the boundary locations rather than determining the quantity of segments. Instead of blindly dividing the frequency axis at rigid geometric intervals, the theoretical partition frequency points ($f_{theo}$) are adaptively translated to their closest physical spectral energy troughs. This guarantees that the total number of parsed segments matches the traditional approach, but their boundaries are physically informed to seamlessly envelop the resonance regions and strictly prevent the truncation of fault features. Mathematically, this nearest-neighbor translation process evaluates the absolute distance between any candidate b and the theoretical node $f_{theo}$. The resulting optimal adaptive boundary, denoted as $b^{*}$, is determined as follows:

(4) $$b^{*}=\underset{b\in \Omega_{\mathrm{bound}}}{\min}\left|b-f_{theo}\right|,$$

where $b^{*}$ represents the updated, physically informed boundary point; $\Omega_{bound}$ denotes the candidate set of local minima (spectral troughs); and $f_{theo}$ is the original geometric boundary defined by the rigid 1/3-binary tree.

**Figure 2:**
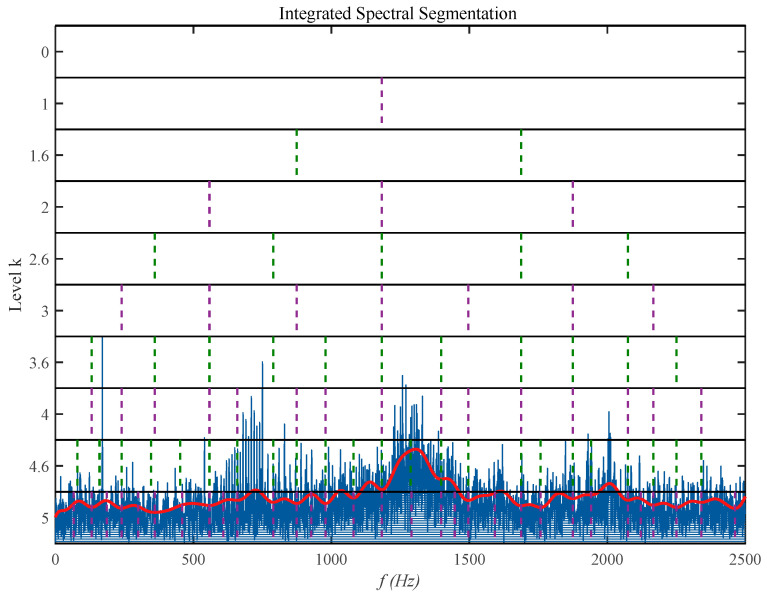
Spectral band decomposition.

As shown by the purple and green dashed lines in Figure 2, all filter cutoff frequencies have been automatically aligned to the troughs (low-energy regions) of the spectral trendline. This paper employs a deterministic adaptive cutoff frequency design for zero-phase IIR Butterworth filters, decomposing signals into a series of narrowband components free from phase distortion. Compared to traditional FIR filtering, this approach not only fully envelops energy-enhanced resonance peaks to prevent feature fragmentation but also delivers high-quality, waveform-accurate input signals for subsequent envelope demodulation through bidirectional filtering (zero-phase).

### 2.2. Targeted Feature Extraction via Adaptive Morphological Consensus

To accurately identify physically meaningful fault harmonics from strong noise interference without requiring prior knowledge of fault characteristics, this paper proposes a feature extraction strategy named Adaptive Morphological Consensus (AMC). Building upon the Candidate Fault Frequencies (CFFs) concept introduced by Cheng et al. [33], this strategy constructs a high-confidence target feature set $\Omega_{target}$ through two stages. It achieves this by deeply exploring the geometric convexity and broadband excitation characteristics of rolling bearing fault signals.

#### 2.2.1. Local Adaptive Morphological Filtering

Spectral peaks generated by fault impacts typically possess specific physical bandwidths and are accompanied by modulated sidebands. Using a fixed peak-search window is highly prone to false detections or missed detections due to scale mismatch. To address this, we define a rotation-correlated structural element scale $\delta_{step}$:

(5) $$\delta_{step}=\max\left(\mathrm{round}\left(\frac{f_{shaft}}{3\cdot \Delta f}\right),1\right),$$

where $\left(f_{shaft}\right)$ denotes the rotational frequency, and (Δf) represents the frequency resolution. This scale provides a physical benchmark for morphological filtering. The structural element scale ($\delta_{step}$) decisively impacts the diagnostic outcome. An undersized empirical scale is overly sensitive, capturing narrow random noise spikes as valid false peaks. Conversely, an oversized scale risks merging genuine fault harmonics with adjacent modulation sidebands, causing missed detections. By explicitly anchoring the scale to $f_{shaft}/3$ in Equation (5), the AMC strategy ensures the analysis window is strictly narrower than the closest possible physical sideband spacing, yet wide enough to reject isolated noise glitches.

Based on this scale, we designed a Bi-directional Differential Operator to rigorously constrain the geometric convexity of spectral peaks. For any frequency (f) and its corresponding squared envelope spectral amplitude $\left(S\left(f\right)\right)$, we define the left gradient $\left(\nabla S_{L}\right)$ and right gradient $\left(\nabla S_{R}\right)$:

(6) $$\left\{\begin{array}{c} \nabla S_{L}(f)=S(f)-S(f-\delta_{\mathrm{step}})\\ \nabla S_{R}(f)=S(f)-S(f+\delta_{\mathrm{step}}) \end{array}\right..$$

Only when a frequency point simultaneously satisfies the bidirectional positive gradient condition (i.e., $\nabla S_{L}>0\wedge \nabla S_{R}>0$) is it classified as a valid morphological peak and recorded by the binary mask M(l,f). This process essentially performs a nonlinear morphological high-hat transformation, effectively stripping away slow-changing background trends and narrowband random glitches.

#### 2.2.2. Global Cross-Band Spectral Consensus

According to the principles of mechanical dynamics, short-duration impacts caused by localized bearing damage manifest as pronounced broadband characteristics in the frequency domain. That is, a single impact simultaneously excites multiple inherent resonance bands within the system. Consequently, the true fault characteristic frequency should not be confined to a single narrowband but should exhibit cross-band recurrence across multiple sub-bands within the adaptive filter bank. In contrast, random noise typically appears only sporadically within specific local frequency bands.

Based on this mechanism, we perform hierarchical accumulation of morphological masks M(l,f) across all subbands to compute the spectral consensus statistic V(f):

(7) $$V\left(f\right)=\sum_{l=1}^{l_{total}}M\left(l,f\right).$$

In Equation (7), $V\left(f\right)$ quantifies the coherent amplitude of frequency f across the entire time–frequency plane. In this case, l denotes the index of the specific sub-band node within the adaptive filter bank, ranging from 1 to the total number of decomposed nodes $l_{total}$. This statistic acts as a physically weighted “voting mechanism,” significantly suppressing false peaks that exhibit local convexity but lack global consistency. Ultimately, the algorithm selects the top D frequencies with the highest consensus based on V(f), forming the target feature set $\Omega_{target}$ for subsequent metric calculations. In this paper, we set D to 10.

### 2.3. Optimal Band Selection Based on WLER Metrics

To quantify the richness of fault features within each sub-band, we propose the Weighted Logarithmic Energy Ratio (WLER) metric. This metric integrates concepts of energy concentration and information entropy.

First, calculate the normalized energy ratio $R\left(f_{i}\right)$ for each candidate frequency $f_{i}\in \Omega_{CFF}$:

(8) $$R\left(f_{i}\right)=\frac{E\left(f_{i}\right)}{{\bar{E}}_{total}},$$

where $E\left(f_{i}\right)$ denotes the energy of the candidate frequency point, and ${\bar{E}}_{total}$ represents the average energy of the entire SES. The WLER metric is defined as follows:

(9) $$\mathrm{WLER}=\frac{1}{D}\sum_{i=1}^{D}\left[R\left(f_{i}\right)\cdot \ln\left(R\left(f_{i}\right)\right)\right].$$

The logarithmic term $\ln\left(R\left(f_{i}\right)\right)$ serves as a nonlinear weighting function with the following properties:

(1) Noise Suppression: For noise components with energy below the average level (i.e., R<1), this term returns negative values, penalizing the overall score.
(2) Feature Enhancement: For fault harmonics with energy significantly exceeding the background (i.e., $E\left(f_{i}\right)\gg {\bar{E}}_{total}$), this term yields substantial positive values, providing exponential rewards to the subband.

The WLER metric introduces an AMC strategy, transforming the originally noise-pulse-sensitive “absolute blind state” into a “semi-blind state” optimization mechanism capable of identifying cyclostable features. Conceptually distinct from traditional negative entropy approaches focused on global spectral statistics, WLER employs a nonlinear logarithmic ratio architecture based on core feature energy versus total energy. This architecture penalizes low-energy noise through logarithmic terms while implementing “exponential” rewards for highly coherent fault harmonics. This enhancement offers dual advantages: it effectively suppresses interference from non-stationary random impacts while significantly amplifying the distinction between faint fault signatures and background noise—all without requiring prior fault knowledge. This ensures precise locking onto the optimal demodulation band even under extremely low SNR conditions.

## 3. Algorithm Implementation Process

**Figure 3:**
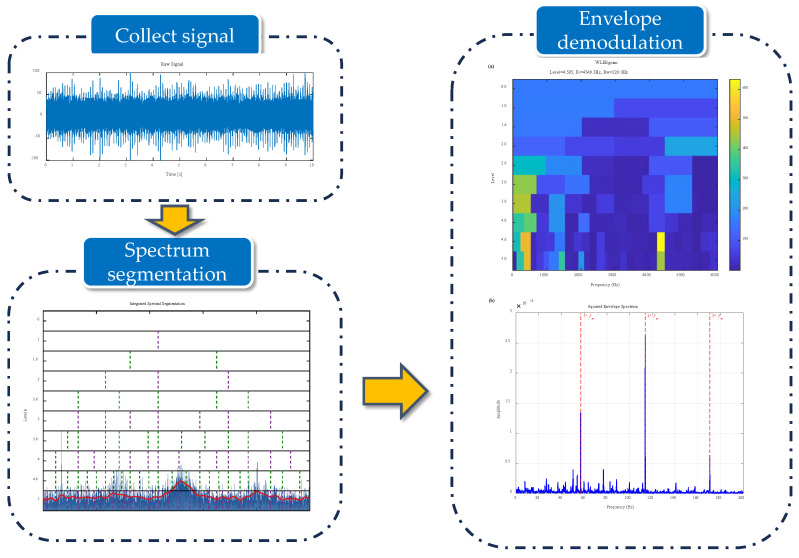
The flowchart of WLERgram.

Based on the above discussion, this section introduces the WLERgram algorithm workflow as shown in Figure 3, which primarily consists of five steps:

(1) Step 1: Reconstruct spectral trends using regularized Fourier series, identifying energy troughs as natural segmentation boundaries;
(2) Step 2: Construct an adaptive 1/3-binary tree filter bank using a nearest-neighbor matching strategy to obtain a series of distortion-free narrowband signals;
(3) Step 3: Perform envelope demodulation on each node signal to compute the SES;
(4) Step 4: Apply the AMC strategy, combining speed-adaptive masking with global spectral consensus to precisely pinpoint target features;
(5) Step 5: Optimize the best frequency band based on the WLER metric to achieve fault diagnosis.

## 4. Simulation Analysis

To verify the effectiveness of the proposed method in extracting bearing fault features under strong background noise and complex interference environments, a synthetic simulation signal $x\left(t\right)$ was constructed. This signal incorporates bearing outer-ring fault impacts, random impulses, discrete frequency interference, and Gaussian white noise. Considering the influence of load fluctuations and speed slip in actual operating conditions, the simulation signal $x\left(t\right)$ is defined as follows:

(10) $$x\left(t\right)=x_{fault}\left(t\right)+x_{rand}\left(t\right)+x_{inter}\left(t\right)+n\left(t\right).$$

In this formulation, $x_{fault}\left(t\right)$ represents the periodic impulsive components excited by the bearing outer-race fault, while $x_{rand}\left(t\right)$ denotes the non-periodic random impulsive interference. The term $x_{inter}\left(t\right)$ accounts for discrete interference, comprising harmonics of the rotational frequency as well as specific interference frequencies, and $n\left(t\right)$ signifies the additive Gaussian white noise.

### 4.1. Failure Impact Model

The simulated bearing outer-race fault signal, $x_{fault}\left(t\right)$, incorporates the effects of random jitter caused by rolling element slip and amplitude modulation induced by load fluctuations. Its mathematical expression is defined as follows:

(11) $$x_{fault}\left(t\right)=\sum_{i=1}^{N}A_{i}\cdot M\left(t\right)\cdot h\left(t-T_{i}-\tau_{i}\right).$$

In the equation, $A_{i}$ represents the impact amplitude; $T_{i}$ denotes the theoretical occurrence time of the i-th impact, determined by the fault characteristic frequency $f_{BPFO}$; $\tau_{i}$ is a random variable following a uniform distribution, used to simulate the random slip effect of ball bearings; M(t) is the load modulation function, simulating the influence of low-frequency load fluctuations on impact amplitude:

(12) $$M\left(t\right)=1+0.3\sin\left(2\pi f_{mod}t\right),$$

where $f_{mod}$ is set to 5 Hz. The impulse response function h(t) employs a double exponential decay model to simulate a more realistic vibration response:

(13) $$h\left(t\right)=\left(e^{-\zeta t}-e^{-2\zeta t}\right)\sin\left(2\pi f_{d}t\right).$$

In the equation, ζ is the damping coefficient, and $f_{d}$ is the system’s natural frequency (resonance frequency) excited by the fault, set to 3500 Hz.

### 4.2. Interference and Noise Model

To increase diagnostic difficulty, complex interference components $x_{inter}\left(t\right)$ are introduced into the analog signal. These include low-order harmonics of the rotation frequency $f_{r}$ and fixed-frequency electromagnetic interference independent of the rotation frequency:

(14) $$x_{inter}\left(t\right)=\sum_{k=1}^{3}C_{k}\sin\left(2\pi kf_{r}t+\phi_{k}\right)+A_{disc}\sin\left(2\pi f_{disc}t\right).$$

In the equation, $f_{disc}$ represents a fixed-frequency disturbance at 1200 Hz. Additionally, the random impact component $x_{rand}\left(t\right)$ is set to excite a high-frequency resonance at 6000 Hz, which differs significantly from the fault resonance frequency (3500 Hz). This configuration aims to test the algorithm’s ability to select the appropriate frequency band under multi-resonance disturbance conditions.

Finally, Gaussian white noise n(t) is added to the synthesized signal to achieve a SNR of −15 dB, simulating harsh operating conditions where strong noise dominates.

**Figure 4:**
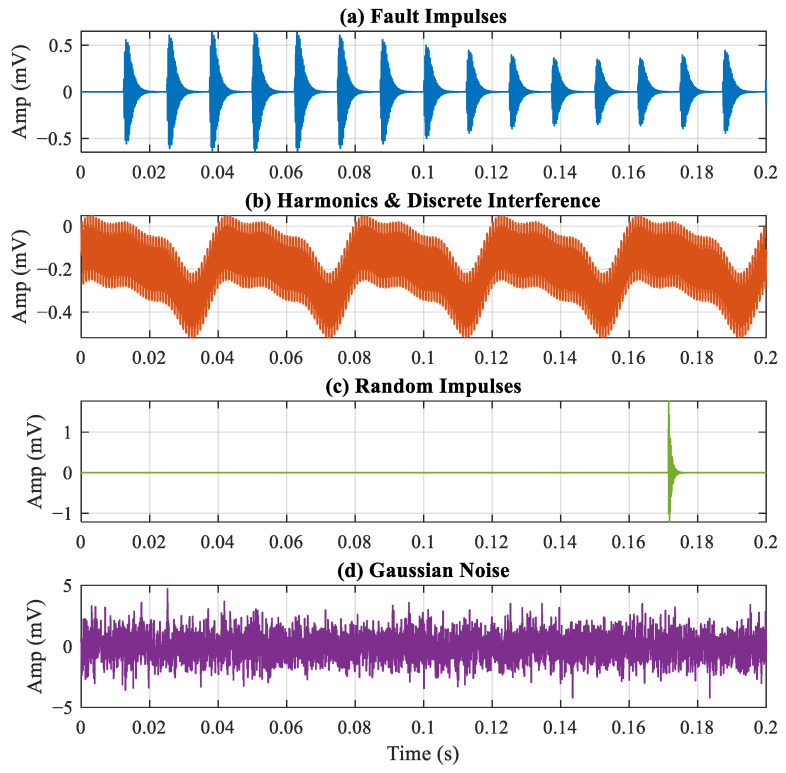
Simulation result.

**Figure 5:**
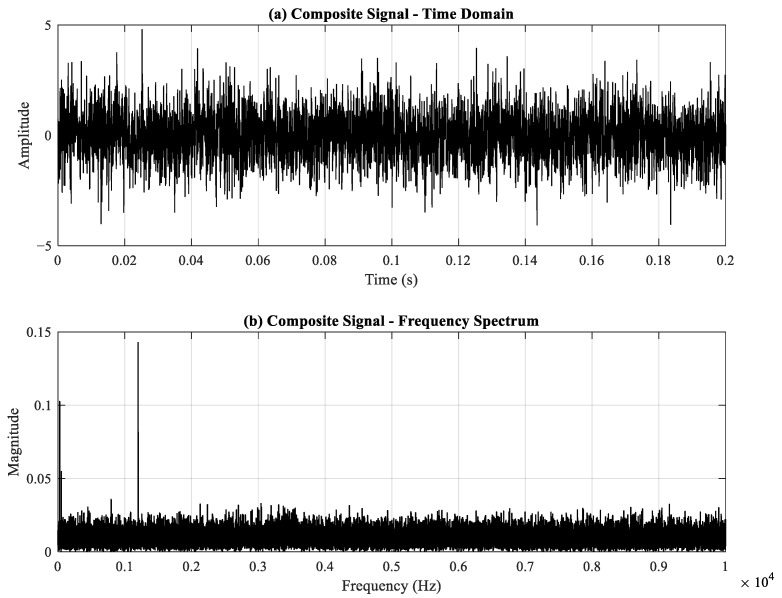
Simulated signal: (**a**) time-domain waveform, (**b**) spectrum.

**Figure 6:**
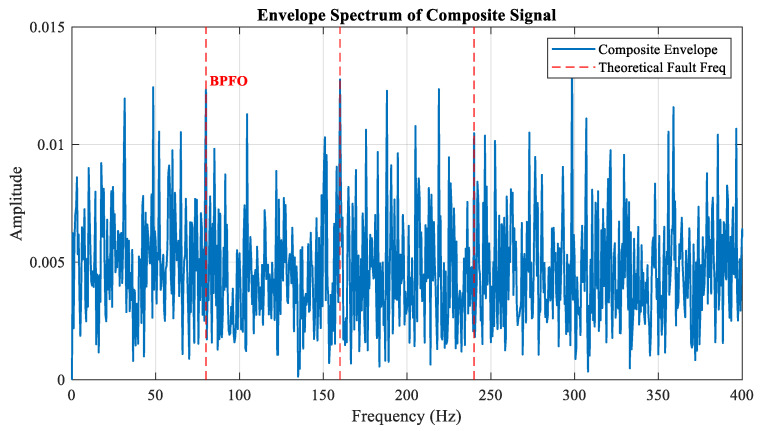
ES of the simulated signal.

The specific parameters are summarized in Table 1. The corresponding simulation results are shown in Figure 4. The synthesized signal consists of four components: periodic impact components triggered by outer ring bearing faults, non-periodic random impact disturbances, discrete frequency components, and Gaussian white noise. Figure 5a,b display the time-domain and frequency-domain plots of the synthesized signal. As shown in Figure 6, the ES of the simulated signal makes it difficult to distinguish the fault frequency. The simulated FCF is 80 Hz, indicated by the red dashed line.

**Figure 7:**
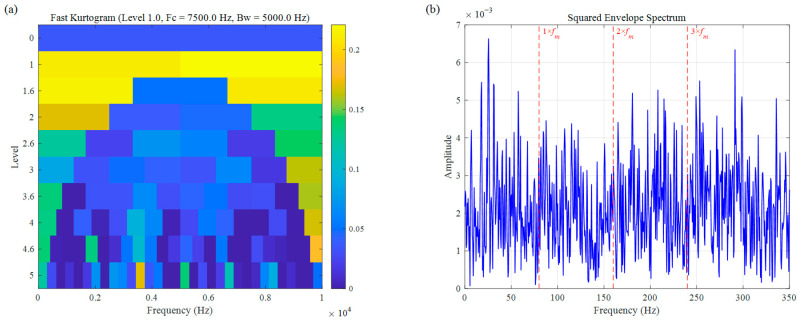
The result of the simulation signal: (**a**) Fast Kurtogram, (**b**) SES.

The Kurtogram is a widely utilized benchmark for bearing fault diagnosis and was originally employed to analyze simulation signals. However, as illustrated in Figure 7, the results indicate that the high sensitivity of kurtosis to random impulses causes the ODFB to be centered at 7500 Hz with a bandwidth of 5000 Hz. This frequency band primarily covers the high-frequency resonance region excited by random impulses within the simulated signal. Because the kurtosis metric is extremely sensitive to sparse, large-amplitude random pulses, the algorithm is “misled” toward non-fault-related regions. Consequently, effective fault characteristic frequencies cannot be extracted from its corresponding SES (Figure 7b).

**Figure 8:**
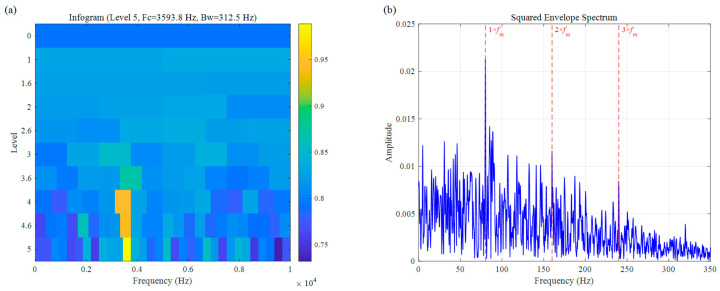
The result of the simulation signal: (**a**) Infogram, (**b**) SES.

As shown in Figure 8, the entropy-based Infogram positions the ODFB at $F_{c}=3593.8$ Hz with a bandwidth of $B_{w}=312.5$ Hz. Although this center frequency is close to the actual fault resonance frequency (3500 Hz), the method’s limited anti-interference capability under strong noise conditions results in significant residual noise within the selected band. Consequently, while weak characteristic frequencies are visible in the SES (Figure 8b), the spectral lines are obscured by clutter, leading to a relatively low SNR.

**Figure 9:**
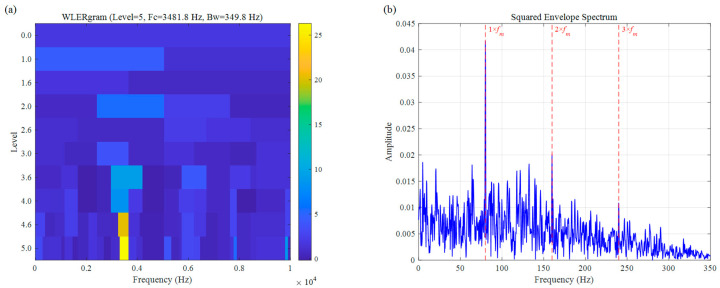
The result of the simulation signal: (**a**) WLERgram, (**b**) SES.

In a complex synthetic simulation environment, WLERgram demonstrates superior capabilities in frequency band localization and feature extraction. By reconstructing the spectral trend, this method overcomes the truncation limits of traditional fixed-band partitioning on resonance energy. Furthermore, the WLER indicator deeply integrates the AMC feature extraction strategy with a nonlinear logarithmic penalty mechanism, enabling “exponential-level” rewards for fault harmonics while suppressing noise components to achieve feature enhancement without prior knowledge. As shown in Figure 9a, the identified ODFB has a center frequency of 3481.8 Hz and a bandwidth of 349.8 Hz. This result aligns closely with the preset fault resonance frequency (3500 Hz), and the selected bandwidth is highly compact, effectively isolating adjacent random impulsive interference. As illustrated in the SES of Figure 9b, the fault characteristic frequency $f_{BPFO}$ (80 Hz) and its second and third harmonics are clearly visible, with amplitudes significantly exceeding the background noise floor. Comparative analysis with Figure 7 and Figure 8 reveals that WLERgram not only successfully avoids the random impulse regions that mislead the Kurtogram, but also achieves a significantly higher level of spectral cleanliness than the Infogram.

**Figure 10:**
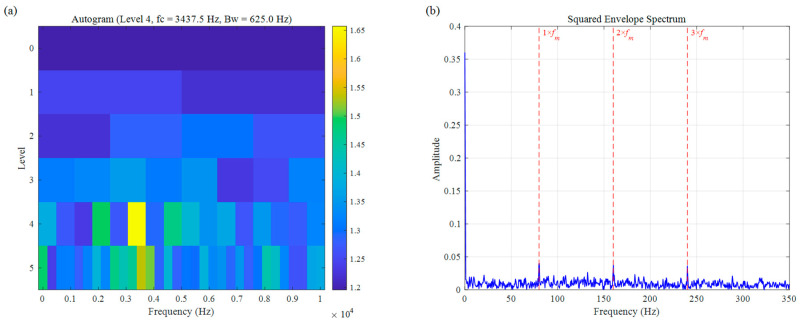
The result of the simulation signal: (**a**) Autogram, (**b**) SES.

**Figure 11:**
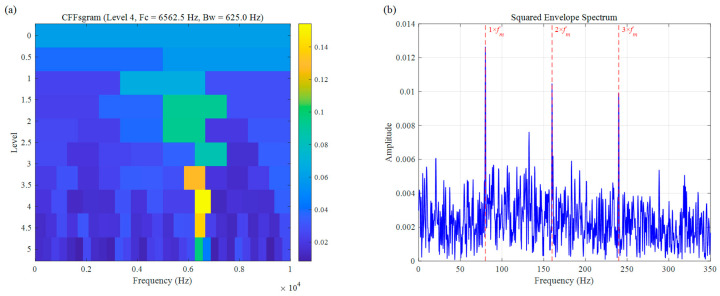
The result of the simulation signal: (**a**) CFFsgram, (**b**) SES.

For a more comprehensive comparison, we also assessed the performance of two other established bearing fault diagnosis approaches: Autogram and CFFsgram. All methods used the same decomposition level of 5, with results shown in Figure 10 and Figure 11. Autogram located a center frequency at 3437.5 Hz ($B_{w}=625.0$ Hz). Although this value lies close to the actual fault resonance band, the method struggles with accuracy under speed fluctuations, which blur the autocorrelation features and lead to suboptimal band selection. In the squared envelope spectrum (Figure 10b), the fault signatures remain weak and unclear, seriously limiting diagnostic reliability. CFFsgram, on the other hand, identified a center frequency of 6562.5 Hz ($B_{w}=625.0$ Hz)—far removed from the true resonance around 3500 Hz. It mistakenly captured a band dominated by random impulse responses instead, leaving almost no visible fault information in the spectrum of Figure 11b.

To provide a comprehensive comparison, the diagnostic signal-to-noise ratio ($SNR_{d}$) was utilized to quantitatively analyze the diagnostic effectiveness of each method, defined as:

(15) $$SNR_{d}=10\log_{10}\left(\frac{\sum_{n=1}^{N}S\left(n\cdot f_{m}\right)}{\sum S\left(f\right)-\sum_{n=1}^{N}S\left(n\cdot f_{m}\right)}\right),$$

where $S\left(f\right)$ represents the amplitude sequence of the Squared Envelope Spectrum (SES), $f_{m}$ denotes the theoretical fault characteristic frequency ($f_{BPFO}$ in this section), n is the harmonic order index, and N is the total number of considered harmonics (set to N=3 in this study). A higher $SNR_{d}$ value indicates more prominent fault features and lower residual background noise.

Table 2 demonstrates that WLERgram achieves the highest $SNR_{d}$ of −10.4958 dB, significantly outperforming the comparative methods. This quantitative advantage stems from the nonlinear mapping of the WLER indicator, which effectively suppresses the energy of non-Gaussian outliers that mislead the Fast Kurtogram ($SNR_{d}$: −30.21 dB). While CFFsgram and Infogram show secondary performance, they remain constrained by rigid frequency partitioning. In contrast, WLERgram’s alignment of filter boundaries with the spectral energy topology ensures the physical integrity of fault resonance bands. The substantial $SNR_{d}$ margin confirms that the synergy between trend-based segmentation and WLER-based enhancement provides superior robustness in isolating weak fault signatures from dominant complex interference.

### 4.3. Quantitative Robustness Analysis Under Varying SNR Levels

**Figure 12:**
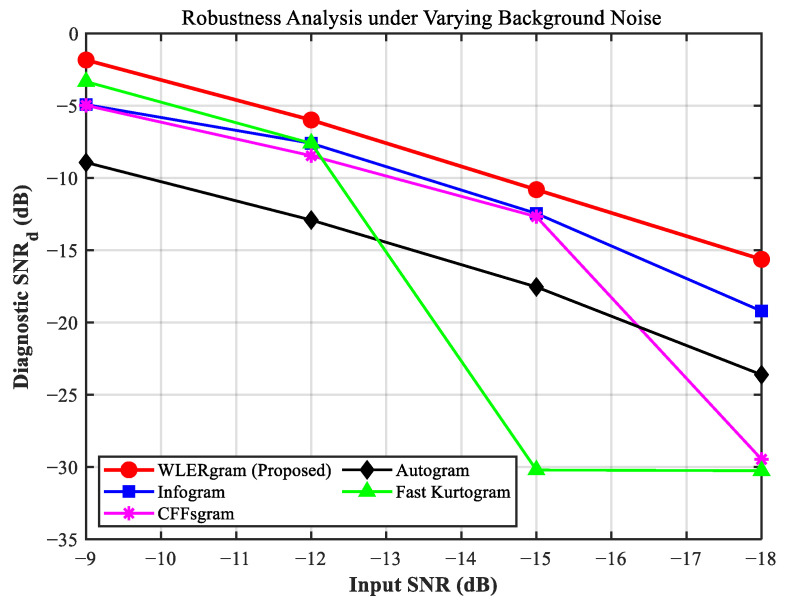
Comparison of diagnostic $SNR_{d}$ among different methods under varying input SNR levels.

As illustrated by the quantitative test in Figure 12, WLERgram demonstrates an overwhelming performance advantage in heavy noise environments (<−15 dB). When the input SNR deteriorates to the extreme condition of −18 dB, traditional methods (e.g., Fast Kurtogram and CFFsgram) suffer catastrophic breakdowns ($SNR_{d}$ plummeting near −30 dB) due to intense random impulse interference. Conversely, WLERgram exhibits no breakdown point and secures the highest $SNR_{d}$ even at −18 dB, outperforming the closest competitor by nearly 4 dB. This drastic performance gap under extreme conditions definitively proves that the nonlinear mechanism of the WLER indicator effectively immunizes against heavy background noise, ensuring a superior feature extraction capability.

Compared to state-of-the-art (SOTA) methodologies, WLERgram achieves a highly competitive balance between computational efficiency and diagnostic robustness. While it introduces a minor computational overhead compared to ultra-fast rigid baselines (e.g., Fast Kurtogram) due to trend reconstruction, this is a necessary trade-off to prevent total failure under heavy noise. Furthermore, WLERgram effectively alleviates the significant computational burden associated with non-downsampling transformations or complex iterative optimization, which is common in certain state-of-the-art methods such as Autogram and CTIgram. Compared to recent adaptive state-of-the-art methods such as CFFsgram, WLERgram maintains comparable computational efficiency. By utilizing closed-form regularized least-squares solutions for spectral fitting and employing highly vectorized operations to implement the AMC strategy, it ensures manageable computational costs while offering significantly stronger noise robustness.

## 5. Experimental Verification

This section demonstrates the effectiveness of the proposed WLERgram through analysis of inner-race fault signals from the CWRU dataset and rolling-element fault signals from the UORED dataset. Comparative evaluations against Fast Kurtogram, Infogram, Autogram, and CFFsgram further reveal the clear advantages of the proposed approach in accurately identifying fault-related resonance bands and extracting prominent fault signatures.

### 5.1. The Rolling Element Failure Signal of CWRU

**Figure 13:**
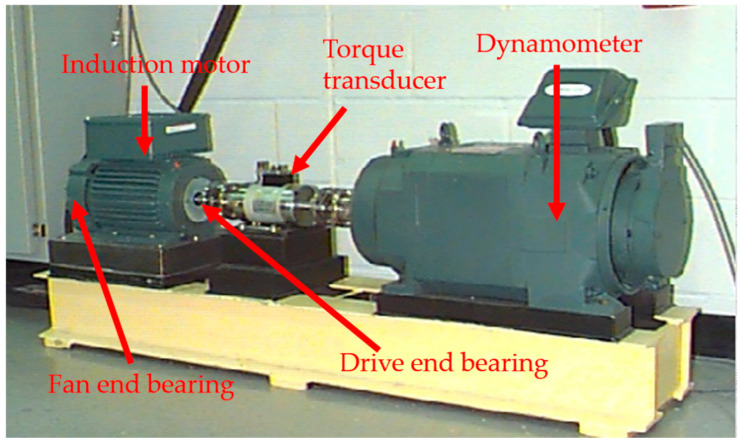
Case Western reserve university test rig.

**Figure 14:**
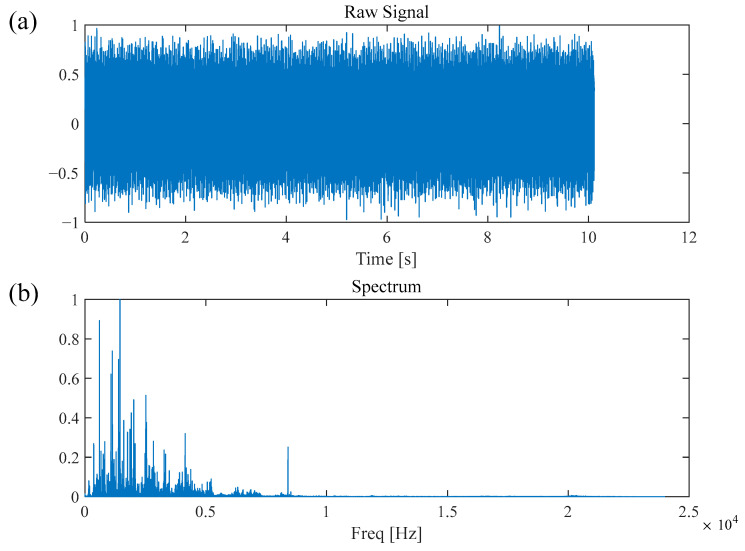
Bearing inner race fault signal of CWRU: (**a**) time-domain waveform, (**b**) Fourier spectrum.

In this section, the fault diagnosis performance of the WLERgram method is validated using the publicly available dataset from CWRU [34]. As shown in Figure 13, the experimental platform primarily consists of a 2-horsepower Reliance electric motor, with the output shaft connected to a torque sensor and an encoder. The vibration signal sampling frequency was set to 48 kHz. This study analyzed the vibration signal from a fan-end bearing (model 6205-2RS JEM, SKF, Gothenburg, Sweden) with a 0.007-inch defect on the inner race. The measured shaft rotational frequency (RF) was approximately 30 Hz, corresponding to a theoretical Ball Pass Frequency on the Inner race (BPFI) of 157 Hz. The time-domain waveform and the corresponding Fourier spectrum of the fault signal are illustrated in Figure 14. As observed, the resonance bands induced by the inner-race fault are difficult to identify directly within the Fourier spectrum.

**Figure 15:**
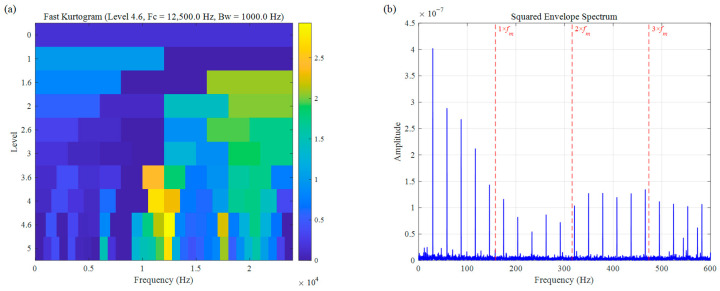
Bearing inner race fault signal of CWRU: (**a**) Fast Kurtogram, (**b**) SES.

First, the Fast Kurtogram was employed to analyze the signal. As illustrated in Figure 15, the kurtosis metric exhibits extreme sensitivity to random impulses, causing the Fast Kurtogram to erroneously locate the center frequency at 12,500 Hz ($B_{w}$ = 1000 Hz) within a high-frequency noise region. Due to its failure to bypass non-fault-related interference, the resulting SES is dominated by cluttered noise, with the 157 Hz characteristic frequency and its harmonics being completely lost. This demonstrates that the Fast Kurtogram is highly susceptible to failure when processing real-world signals containing intense random impulses.

**Figure 16:**
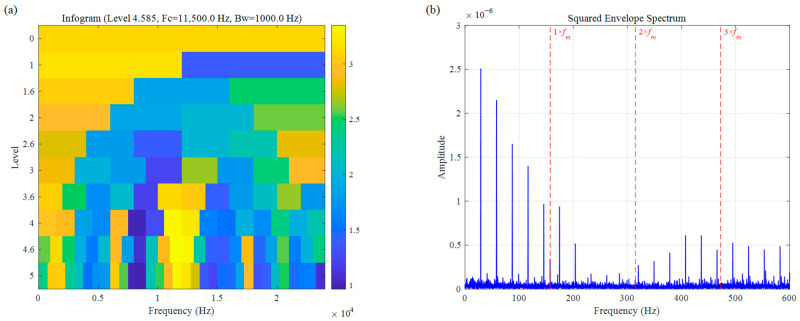
Bearing inner race fault signal of CWRU: (**a**) Infogram, (**b**) SES.

The Infogram is similarly compromised by high-frequency noise interference. As illustrated in Figure 16, the method is drawn toward high-frequency components, with the selected frequency band centered near 11,500 Hz. Although traces of the fault frequency can be faintly observed in the SES, the characteristic amplitudes are extremely low and obscured by significant environmental interference. Consequently, the overall SNR is poor, failing to meet the requirements for precise fault diagnosis.

**Figure 17:**
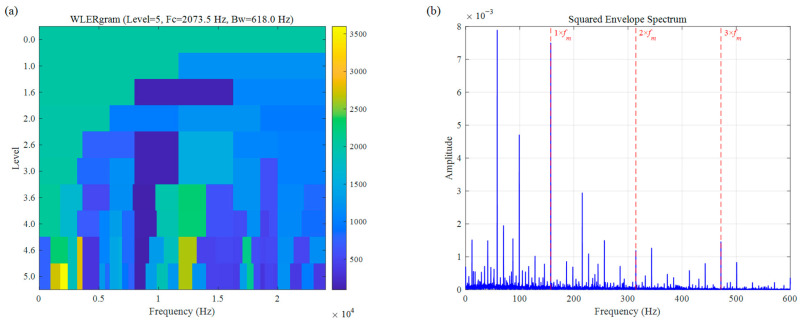
Bearing inner race fault signal of CWRU: (**a**) WLERgram, (**b**) SES.

As illustrated in Figure 17, WLERgram demonstrates exceptional frequency band localization accuracy and feature enhancement capabilities when processing real-world CWRU signals. Attributed to the adaptive segmentation of the spectral trend and the precise targeting of the AMC strategy, this method successfully bypasses high-frequency interference to identify the ODFB in the low-frequency region (Level 5, $F_{c}=2073.5$ Hz, $B_{w}=618.0$ Hz). In the SES of Figure 17b, the rolling-element fault characteristic frequency $f_{m}$ and its harmonics ($2f_{m},3f_{m}$) are clearly prominent, while background noise is effectively suppressed.

**Figure 18:**
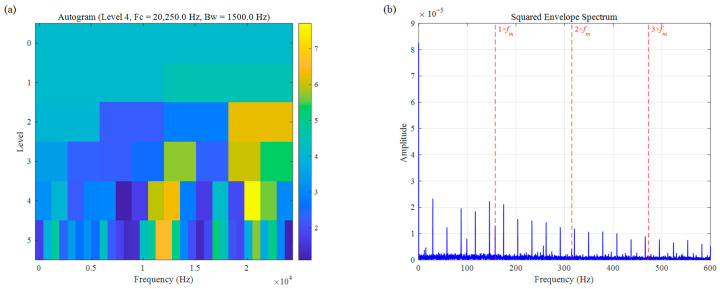
Bearing inner race fault signal of CWRU: (**a**) Autogram, (**b**) SES.

**Figure 19:**
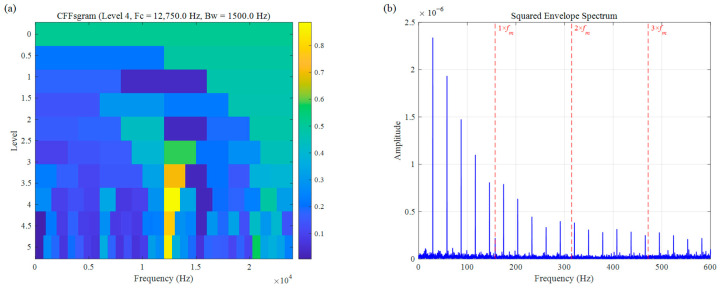
Bearing inner race fault signal of CWRU: (**a**) CFFsgram, (**b**) SES.

In the context of processing complex practical signals from the CWRU dataset, as shown in Figure 18 and Figure 19, both Autogram and CFFsgram failed to accurately localize the resonance bands. Under the joint interference of intense background noise and random impulses, the optimal frequency band identified by Autogram shifted to an ultra-high frequency region of 20,250 Hz; this is primarily because its reliance on the squared envelope autocorrelation metric makes it highly susceptible to feature blurring in low SNR conditions, causing it to lock onto frequency segments with high noise energy. Similarly, CFFsgram exhibited a positioning error, with its center frequency incorrectly identified at 12,750 Hz. Although it considers candidate fault frequencies, the lack of an adaptive spectral trend reconstruction mechanism means its fixed band partitioning tends to dilute the faint resonance energy excited by faults. As evidenced by the squared envelope spectra in Figure 18b and Figure 19b, both results are dominated by chaotic noise components, with the inner-ring fault characteristic frequency $f_{m}$ and its harmonics being completely submerged, demonstrating that these methods lack sufficient robustness and feature extraction capability when handling complex real-world signals with broadband background noise.

Table 3 shows that WLERgram is the only method yielding a favorable $SNR_{d}$ of −4.4236 dB, while the counterparts remain significantly lower (e.g., Fast Kurtogram at −21.31 dB). This performance gap highlights WLERgram’s superior efficacy in isolating faint fault signatures amidst the dense background noise typical of industrial environments. The consistent $SNR_{d}$ lead indicates that the adaptive boundary alignment effectively preserves the underlying fault periodicity, whereas traditional methods struggle with noise-induced band-selection errors. These results underscore WLERgram’s robust engineering utility in processing non-stationary, low-SNR experimental signals.

### 5.2. Rolling Body Fault Signal at the UORED

**Figure 20:**
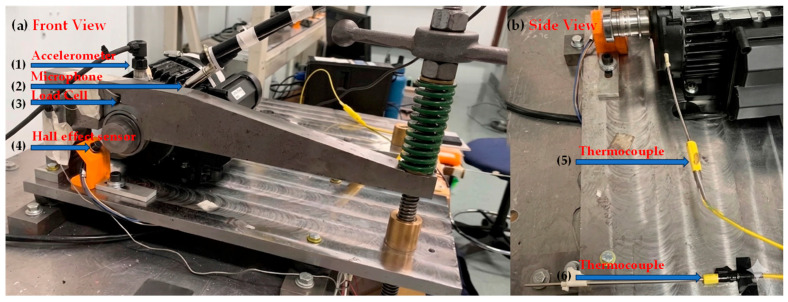
UORED-VAFCLS test rig set up in operation: (**a**) Front view, (**b**) side view.

**Figure 21:**
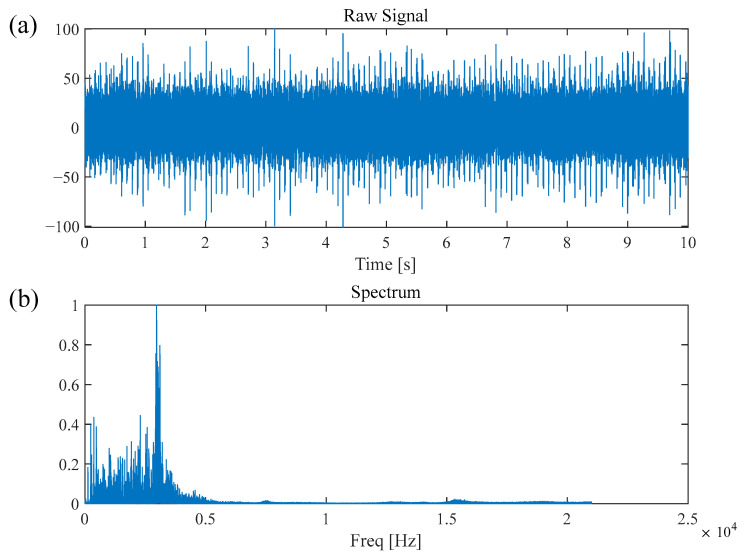
Rolling element fault signal of UORED: (**a**) time-domain waveform, (**b**) Fourier spectrum.

The validation data for this study was sourced from the University of Ottawa (UODS-VAFDC) dataset [35]. The experimental platform was driven by a Lafert LM-71-S4 (Lafert Group, San Donà di Piave, Italy) motor operating at a constant speed of 1750 RPM. As illustrated in Figure 20, the test rig [36] is equipped with six types of sensors: (1) an accelerometer, (2) a microphone, (3) a force sensor, (4) a Hall effect sensor, (5) a motor temperature thermocouple, and (6) an ambient temperature thermocouple. For rolling-element fault detection, vibration data from a FAFNIR 203KD (Fafnir Bearing Company, New Britain, CT, USA) deep-groove ball bearing under no-load (0 N) conditions (samples B-11 to B-15) was selected. The theoretical fault characteristic frequency was approximately 116 Hz. The vibration signals were captured using a PCB 623C01 (PCB Piezotronics, Depew, NY, USA) accelerometer at a high sampling rate of 42 kHz, ensuring the effective capture of weak rolling-element fault impulses and their higher-order harmonics. The time-domain waveform and corresponding Fourier spectrum of the fault signal are shown in Figure 21; it is evident that the resonance bands induced by the rolling-element fault are difficult to discern directly within the Fourier spectrum.

**Figure 22:**
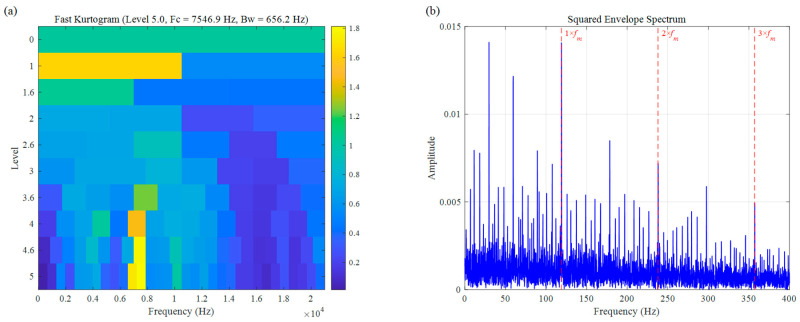
Rolling element fault signal of UORED: (**a**) Fast Kurtosis, (**b**) SES.

First, the diagnostic results of the Fast Kurtogram are presented in Figure 22. Due to the faint nature of rolling-element fault impulses and their inherent random slip characteristics, the kurtosis metric is highly susceptible to interference from random components within the background noise. This sensitivity caused the Fast Kurtogram to erroneously locate the optimal frequency band at 7546.9 Hz ($B_{w}$ = 656.2 Hz). Although the fault characteristic frequency $f_{m}$ at 116 Hz and its weak harmonics can be observed in the corresponding SES (Figure 22b), the spectral lines appear extremely cluttered. Significant random interference leads to an elevated noise floor, which obscures the fault characteristic peaks and severely compromises the SNR, rendering an effective diagnosis difficult.

**Figure 23:**
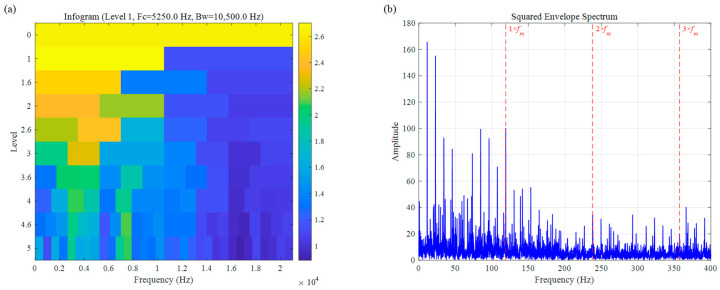
Rolling element fault signal of UORED: (**a**) Infogram, (**b**) SES.

The analytical results for the Infogram are presented in Figure 23. Based on the negative entropy metric, this method tends to favor regions with wider bandwidths; consequently, it identified an optimal center frequency of 5250.0 Hz with a bandwidth as high as 10,500.0 Hz (Level 1). This excessively wide frequency band introduces a significant amount of out-of-band noise. As a result, although faint frequency components are visible in Figure 23b, the SNR remains extremely low, making it impossible to clearly distinguish the characteristic harmonics of the rolling-element fault.

**Figure 24:**
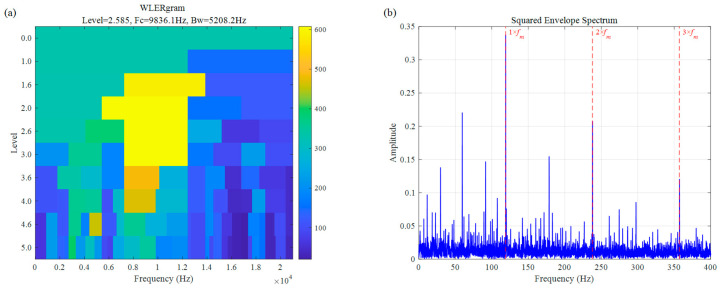
Rolling element fault signal of UORED: (**a**) WLERgram, (**b**) SES.

As illustrated in Figure 24, WLERgram demonstrates significant advantages in feature extraction when dealing with faint and slipping rolling-element faults under no-load conditions. As shown in Figure 24a, by utilizing adaptive spectral trend segmentation, the method successfully locks onto a high-frequency resonance band with a center frequency ($F_{c}$) of 9836.1 Hz and a bandwidth of 5208.2 Hz, thereby effectively bypassing the intense mechanical interference present in lower frequency ranges. In the corresponding SES (Figure 24b), the fault characteristic frequency ($f_{m}$ at 116 Hz) and its high-order harmonics ($2f_{m},3f_{m}$) exhibit exceptionally prominent amplitudes. Furthermore, due to the nonlinear penalty mechanism of the WLER indicator against noise components, the spectral background remains remarkably clean.

**Figure 25:**
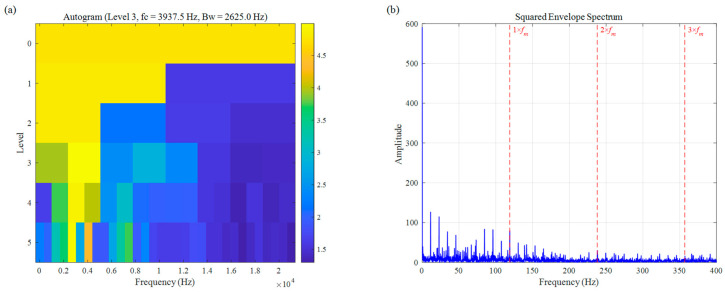
Rolling element fault signal of UORED: (**a**) Autogram, (**b**) SES.

**Figure 26:**
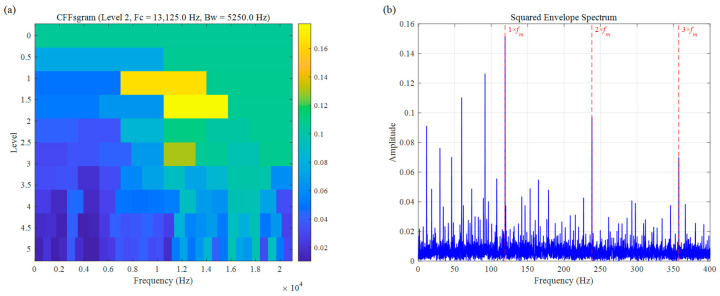
Rolling element fault signal of UORED: (**a**) CFFsgram, (**b**) SES.

In the diagnosis of weak rolling-element faults under no-load conditions, the performance of both Autogram (Figure 25) and CFFsgram (Figure 26) is suboptimal. As illustrated in Figure 25a, the center frequency selected by Autogram is located at 3937.5 Hz; however, its reliance on the autocorrelation kurtosis metric makes it highly susceptible to interference from random components in the background noise under extremely low SNR, leading to a deviation in the band selection logic. Meanwhile, CFFsgram (Figure 26) locked onto a high-frequency band at 13,125.0 Hz. Although this method utilizes candidate fault frequency information, the absence of an adaptive spectral trend partitioning mechanism causes its rigid boundaries to dilute the faint resonance energy within the strong background noise. Observation of the SES in Figure 25b reveals that no significant characteristic peaks appear at the fault characteristic frequency $f_{m}$ (116 Hz) or its harmonics. Similarly, in Figure 26b, a vast number of spectral lines caused by random interference components render the fault characteristic peaks indistinct. These results substantiate a severe lack of feature extraction capability and noise robustness in traditional methods when processing such low-energy impulsive signals.

Table 4 summarizes the $SNR_{d}$ comparison, where WLERgram achieves the highest value of −13.9906 dB, consistently surpassing the comparative methods. This quantitative outcome aligns with the theoretical expectation that WLERgram’s adaptive boundary alignment effectively preserves fault-specific harmonics—components that are typically attenuated by rigid filtering processes. The superior $SNR_{d}$ performance under real-world operating conditions further substantiates that the proposed nonlinear mapping mechanism provides a more discriminative evaluation of weak fault components than traditional statistical indicators. These results validate WLERgram as a robust solution for isolating incipient signatures from the dominant interference inherent in practical engineering datasets.

## 6. Conclusions

This paper proposes a novel rolling bearing fault diagnosis method, termed WLERgram, aimed at overcoming the challenges of extracting weak early-stage fault features under intense background noise and random impulsive interference. The method introduces regularized Fourier series to reconstruct the spectral trend, utilizing the natural “troughs” of the energy distribution to achieve adaptive spectrum segmentation. This effectively overcomes the truncation effect of traditional fixed boundaries (such as the 1/3-binary tree) on resonance bands. Building upon this, an AMC strategy correlated with rotational speed is combined with a WLER indicator to construct a feature enhancement and band optimization framework that requires no prior fault knowledge. Through the deep integration of physical and statistical features, WLERgram ensures the precise capture and complete preservation of fault impulse sequences in low SNR environments.

Systematic validation through synthetic simulation signals and dual experimental datasets (CWRU and UORED) confirms the clear superiority of the proposed WLERgram method. Under harsh operating conditions dominated by strong background noise and random interference, traditional approaches reveal distinct limitations: Fast Kurtogram frequently selects erroneous bands due to its vulnerability to non-Gaussian random impulses; Infogram often captures excessively broad frequency ranges, leaving substantial residual noise; Autogram and CFFsgram struggle to reliably locate weak fault features, primarily because they lack an adaptive spectral segmentation strategy. By contrast, WLERgram exhibits outstanding robustness across all tested scenarios, consistently outperforming the comparative algorithms. This stems from its ability to align frequency band selection with physically meaningful spectral trends, combined with the nonlinear penalty mechanism inherent in the WLER indicator, which effectively suppresses non-Gaussian noise while enhancing the extraction of high-order weak harmonics.

Although WLERgram demonstrates superior robustness on simulated and benchmark datasets (e.g., CWRU and UORED), its validation on complex industrial datasets remains a limitation. To address this, future research will focus on deploying the methodology in real-world industrial environments. This includes enhancing its adaptability to time-varying speeds and non-stationary conditions via dynamic spectral trend segmentation, and investigating its integration with lightweight deep learning networks as a high-quality feature preprocessor for end-to-end real-time fault diagnosis and remaining useful life prediction.

## Figures and Tables

**Table 1:** Parameter list for simulation model.

**Fs [kHz]** $f_{s}$	**Speed [RPM]** *n*	**Fault Characteristic Freq** $f_{BPFO}$	**Resonance Frequency** $f_{d}$	**Damping Coefficient** ζ	**Load Modulation Freq** $f_{mod}$
20 kHz	1500	80 Hz	3500 Hz	800	5 Hz
**Random Impulse Resonance** $f_{rand}$	**Discrete Interference** $f_{discrete}$	**Harmonic** $C_{1}$	**Harmonic** $C_{2}$	**Harmonic** $C_{3}$	**SNR [dB]**
6000 Hz	1200 Hz	0.1	0.05	0.03	−15

**Table 2:** Comparison of $SNR_{d}$ values among different methods in simulation.

Methods	WLERgram	Autogram	Infogram	Fast Kurtogram	CFFsgram
**Value**	−10.4958	−17.5437	−15.0165	−30.21	−13.9268

**Table 3:** Comparison of $SNR_{d}$ values among different methods in the CWRU dataset.

Methods	WLERgram	Autogram	Infogram	Fast Kurtogram	CFFsgram
**Value**	−4.4236	−20.5118	−25.0698	−21.31	−29.1689

**Table 4:** Comparison of $SNR_{d}$ values among different methods in UORED.

Methods	WLERgram	Autogram	Infogram	Fast Kurtogram	CFFsgram
**Value**	−13.9906	−20.5789	−19.7229	−14.32	−17.1781

## Data Availability

The original contributions presented in this study are included in the article. Further inquiries can be directed to the corresponding author.
